# Halogenated dicyanobenzene-based photosensitizer (3DPAFIPN) as a thermally activated delayed fluorescence (TADF) used in gram-scale photosynthesis 3,4-dihydropyrimidin-2-(1*H*)-one/thione derivatives via a consecutive visible-light-induced electron-transfer pathway

**DOI:** 10.3389/fchem.2024.1361266

**Published:** 2024-03-01

**Authors:** Farzaneh Mohamadpour, Ali Mohammad Amani

**Affiliations:** Department of Medical Nanotechnology, School of Advanced Medical Sciences and Technologies, Shiraz University of Medical Sciences, Shiraz, Iran

**Keywords:** dicyanobenzene-based photosensitizer (3DPAFIPN), renewable energy source, visible-light-induced electron-transfer, photosynthesis, 3, 4-dihydropyrimidin-2-(1H)-one/thione derivatives

## Abstract

**Background:** Organic dyes often have shorter lifetimes in the excited state, which is a major obstacle to the development of effective photoredox methods. The scientific community has shown a great deal of interest in a certain class of organic chromophores because of their unique characteristics and effectiveness. One characteristic of the molecules under research is thermally activated delayed fluorescence (TADF), which is only observed in molecules with a tiny energy gap (often less than 0.2 eV) between their lowest two excited states, i.e., singlet excited state (S_1_) and triplet excited state (T_1_). The extended singlet excited states arising from TADF and the simplicity with which their redox potentials may be altered make the isophthalonitrile family of chromophores an attractive option for organic photocatalyst applications.

**Methods:** The Biginelli reaction between *β*-ketoesters, arylaldehydes, and urea/thiourea has been used to build a sustainable technique for the production of 3,4-dihydropyrimidin-2-(1*H*)-one/thione derivatives. In the present study, the development of a green radical synthesis approach for this class of compounds is addressed in depth. As a photocatalyst, a new halogenated dicyanobenzene-based photosensitizer was employed in this study. As a renewable energy source activated by a blue LED, it was dissolved in ethanol, at room temperature in air atmosphere. The primary objective of this research is to employ a novel donor-acceptor (D-A) based on halogenated cyanoarene that is affordable, easily available, and innovative.

**Findings:** The 3DPAFIPN [2,4,6-tris(diphenylamino)-5-fluoroisophthalonitrile] photocatalyst, a thermally activated delayed fluorescence (TADF), induces single-electron transfer (SET) in response to visible light, offering a straightforward, eco-friendly, and highly efficient process. Additionally, we determined the 3,4-dihydropyrimidin-2-(1*H*)-one/thione derivatives turnover frequency (TOF) and turnover number (TON). It has also been demonstrated that gram-scale cyclization is a workable method for industrial purposes.

## Introduction

In recent literature, photoredox catalysis has served as a foundation for novel approaches in organic chemistry (Mohamadpour, 2021a; Mohamadpour, 2023a). The field of photoredox catalysis, which combines metal-promoted reactions with photoredox cycles, is gaining significant attention from both academia and industry (Pinosa et al., 2022). The main focus of research is the use of inexpensive, readily manufactured, and efficient organic dyes to help create novel, powerful, and selective metal-promoted reactions (Gualandi et al., 2021). In this sector, organic dyes must take the place of the commonly employed inorganic complexes that are dependent on Ir(III) and Ru(II). When compared to organic molecules, these compounds are known for their long excited state lifetimes, which may tend toward dynamic quenching. Organic dyes often have shorter lifetimes in the excited state, which is a major obstacle to the development of effective photoredox methods. The scientific community has shown a great deal of interest in a specific class of organic chromophores because of their unique characteristics and effectiveness (Bryden and Zysman-Colman, 2021). One characteristic of the molecules under study is thermally activated delayed fluorescence (TADF), which is only observed in molecules with a tiny energy gap (often less than 0.2 eV) between their lowest two excited states, i.e., S_1_ and T_1_. Under ambient conditions, the molecules under study undergo reverse intersystem crossing (RISC), aided by a thermally activated pathway from the triplet excited state (T_1_) to the singlet excited state (S_1_). This results in a delayed fluorescence phenomenon that is commonly observed in systems similar to this one. The present goal is to combine reduced instruction set computing’s (RISC) exceptional efficiency with fluorescence’s great quantum yield. 2012 saw a significant advancement in the field of organic light-emitting diodes (OLEDs) with the release of a basic work by Adachi (Uoyama et al., 2012). This approach covers the efficient usage of dicyanobenzene molecules with suitable photophysical properties as well as their demonstrated application in OLEDs. Similar TADF chromophores have been used in other domains, such as photocatalysis, since these initial discoveries (Yang et al., 2017; Bryden and Zysman-Colman, 2021). The extended singlet excited states arising from TADF and the simplicity with which their redox potentials may be altered make the isophthalonitrile family of chromophores a viable option for organic photocatalyst applications (Speckmeier et al., 2018). 2,4,6-tris(diphenylamino)-5-fluoroisophthalonitrile (3DPAFIPN) is a chemical that is widely used in a number of visible light-triggered synthetic procedures. Intramolecular cyclizations (Flynn et al., 2020; Wu et al., 2020) and the formation of C–C (Cardinale et al., 2020; Donabauer et al., 2020), N–C (Zhou et al., 2020), and P–C (Rothfelder et al., 2021) bonds (Pinosa et al., 2022) are a few examples of these processes.

Because visible light irradiation has a large energy reserve, is inexpensive, and can be used to access sustainable energy sources, it is considered a reliable method for creating organic compounds (Mohamadpour, 2021b; Mohamadpour, 2021c; Mohamadpour, 2022a).

**FIGURE 1:**
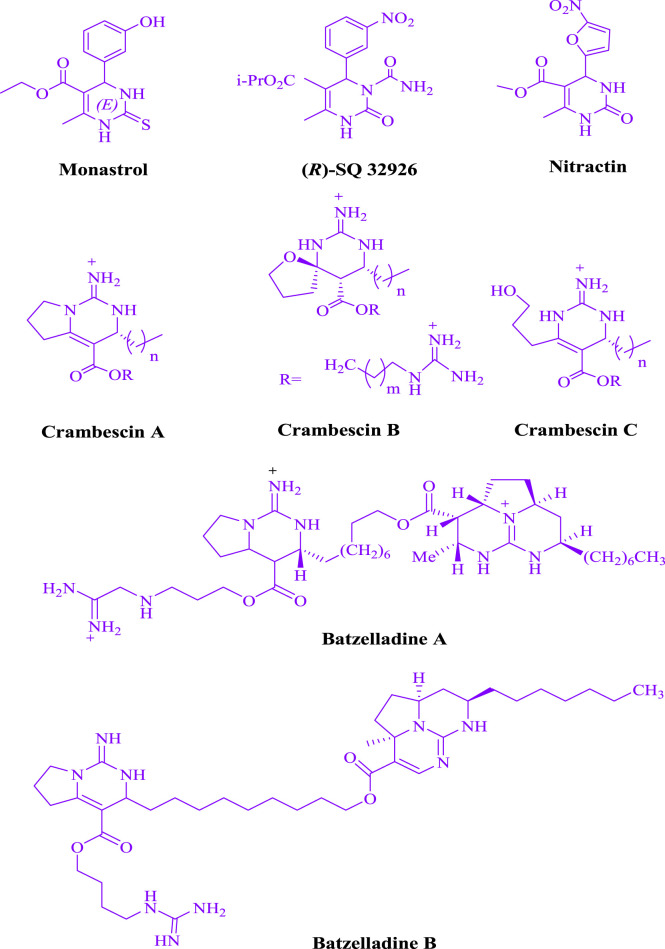
The dihydropyrimidine motifs exhibit pharmacological activity.

It is expected that dihydropyrimidine structures have potent biological and pharmacological effects (Figure 1). Calcium channel blockers, antihypertensive effects (Sujatha et al., 2006), anticancer (Wisen et al., 2008), anti HIV agent (Heys et al., 2000), antibacterial and antifungal (Ashok et al., 2007), antiviral (Hurst and Hull, 1961), antioxidative (Magerramow et al., 2006), and anti-inflammatory (Bahekar and Shinde, 2004).

To produce 3,4-dihydropyrimidin-2-(1*H*)-one/thione derivatives, a number of catalysts are employed, including Na_2_ eosin Y (Mohamadpour, 2022b), copper (II)sulfamate (Liu et al., 2012), bakers, yeast (Kumar and Maurya, 2007), hydrotalcite (Lal et al., 2012), hexaaquaaluminium (III) tetrafluoroborate (Litvic et al., 2010), TBAB (Ahmed et al., 2009), copper (II) tetrafluoroborate (Kamal et al., 2007), [Btto][*p*-TSA] (Zhang et al., 2015), triethylammonium acetate (Attri et al., 2017), saccharin (Mohamadpour et al., 2016), caffeine (Mohamadpour and Lashkari, 2018), zirconium (IV)-salophen perfluorooctanesulfonate (Li et al., 2020), H_3_ [PW_12_O_40_] (Chopda and Dave, 2020), Dioxane-HCl (Choudhare et al., 2021), WSi/A15 (Bosica et al., 2021), H_4_ [W_12_SiO_40_] (V Chopda and Dave, 2020), Zr(H_2_PO_4_)_2_ (KÜÇÜKİSLAMOĞLU et al., 2010), and GO-chitosan (Maleki and Paydar, 2016). Complex procedures, lengthy reaction times, the use of costly chemicals, and lower yields are just a few of the variables that significantly impact the management and disposal of waste. Moreover, it might be challenging to extract homogeneous catalysts from reaction mixtures. Due to our interest in the development of photocatalytic reactions (Mohamadpour, 2022c; Mohamadpour, 2022d; Mohamadpour, 2023b; Mohamadpour, 2023c; Mohamadpour, 2023d), the current work discusses the use of photocatalysts in the synthesis of heterocyclic compounds, emphasizing the use of environmentally acceptable practices. The investigation indicates that using photo-redox catalysts for halogenated organic dyes is also financially feasible. A potent donor-acceptor (D-A) cyanoarene is employed as an efficient organo-photocatalyst by employing the previously outlined technique.

The primary focus of the investigation was 2,4,6-tris(diphenylamino)-5-fluoroisophthalonitrile (3DPAFIPN) because of its exceptional photophysical and photochemical properties. Organic chemists now have access to a wider range of photocatalysts thanks to the development of dicyanobenzene-based photosensitizers, which demonstrate exceptional photoelectric activity and thermally activated delayed fluorescence (TADF).

The current study has investigated 3DPAFIPN, a new halogenated cyanoarene-based donor-acceptor (D-A) photocatalyst that works by a sequence of visible-light-induced electron transfers. The three-condensation domino Biginelli reaction arylaldehydes with urea/thiourea and *β*-ketoesters are used in this procedure. In addition, this process employs blue LED, a sustainable and environmentally friendly energy source, in a room-temperature, ethanol medium as a green solvent. Regardless of the timely and effective completion of all obligations and adherence to the agreed-upon budget.

## Experimental

### General

To find each compound’s melting point, an electrothermal instrument, a 9,100, was employed. ^1^HNMR spectra were collected using Bruker DRX-300 Avance equipment with DMSO-d_6_. Materials and reagents were acquired from Acros, Merck, and Fluka and utilized right away.

### The sustainable method for 3,4-dihydropyrimidin-2-(1H)-one/thione derivatives (4a-t)

**SCHEME 1:**
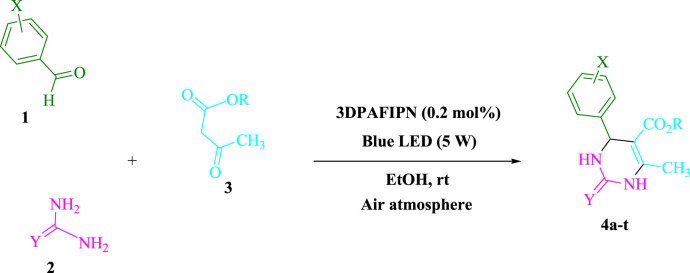
Light-mediated synthesis of 3,4-dihydropyrimidin-2-(1*H*)-one/thione derivatives.

At room temperature in EtOH (3 mL), urea/thiourea (**2**, 1.5 mmol), ethyl/methyl acetoacetate (**3**, 1.0 mmol), and arylaldehyde derivatives (**1**, 1.0 mmol) were stirred with 3DPAFIPN (0.2 mol%) and blue light (5 W) (Scheme 1). We used thin-layer chromatography (TLC) to track the reaction’s development. Without requiring any additional purification procedures, the pure substance was obtained by screening, washing with water and ethanol, and crystallizing the crude solid from ethanol following the reaction. The Supplementary Material file contains a report on spectroscopic data.

## Results and discussion

**FIGURE 2:**
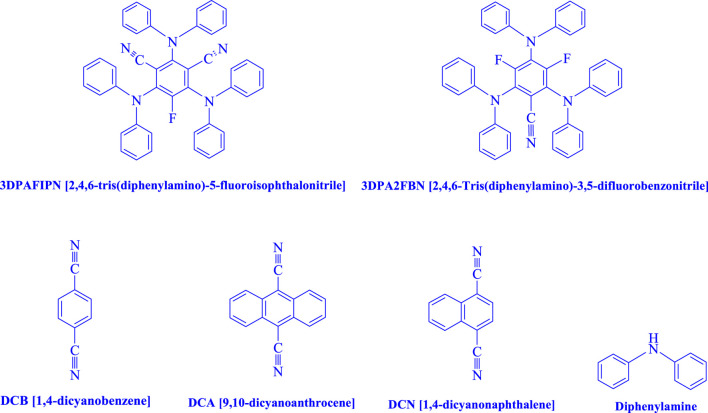
The suitability of the catalyst was evaluated in this study.

The reaction was optimized in the current study using a 3 mL ethanol medium containing 1.0 mmol of benzaldehyde, 1.5 mmol of urea, and 1.0 mmol of ethyl acetoacetate. Without the aid of a photocatalyst, a trace quantity of **4j** was produced for 20 min at room temperature in the presence of 3 mL of EtOH. The pace of reaction was enhanced by the inclusion of photocatalysts. The compounds include 3DPAFIPN, 3DPA2FBN, DCB, DCA, DCN, and diphenylamine, as indicated by the data in Figure 2

The present technique can create **4j** with varying yields. The aforementioned data showed that 3DPAFIPN’s operational efficacy has increased. A reaction with 0.2 mol% 3DPAFIPN yielded a 97% yield, based on the data in Table 1, entry 2. Results for solvent-free conditions, EtOAc, DMSO, toluene, H_2_O, EtOH, MeOH, CHCl_3_, CH_3_CN, THF, and are displayed in Table 2. In the presence of EtOH, the reaction was demonstrated to have a notably enhanced rate and subsequent yield. Based on the data in Table 2, especially entry 5, a 97% yield was achieved. Table 2 lists the light sources that have been used in studies to assess the impact of blue light on production. In the assessment that was conducted without the use of an illumination tool, the **4j** was discovered in extremely little amounts. The results of this investigation demonstrate that 3DPAFIPN and visible light are essential for the effective synthesis of product **4j**. The top designs were determined using blue light-emitting diode (LED) intensities of 3 W, 5 W, and 7 W. The results of the investigation showed that the greatest results were obtained when blue light-emitting diodes (LEDs) with a 5 W power output were used. The outcomes of studies conducted on a range of substrates under optimal conditions are displayed in Table 3; Scheme 1 (and also Supplementary Table S1). The benzaldehyde substituent has no effect on the reaction’s result (Table 3). Both polar and halide substitutions were permitted in this reaction. In the current state of the reaction, reactions involving both electron-donating and electron-withdrawing functional groups are acceptable. Aromatic aldehydes that are *ortho*, *meta*, and *para*-substituted have a very high yield potential. The reactions of methyl and ethyl acetoacetate are comparable. Urea and thiourea have comparable reactivities.

**TABLE 1:** This table optimizes the photocatalyst for the synthesis of **4j** ^a^.

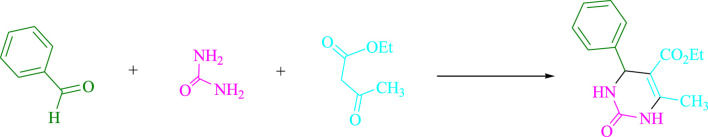				
Entry	Photocatalyst	Solvent (3 mL)	Time (min)	Isolated Yields (%)
1	3DPAFIPN (0.1 mol%)	EtOH	5	84
2	3DPAFIPN (0.2 mol%)	EtOH	5	97
3	3DPA2FBN (0.2 mol%)	EtOH	5	82
4	DCB (0.2 mol%)	EtOH	5	19
5	DCA (0.2 mol%)	EtOH	5	27
6	DCN (0.2 mol%)	EtOH	5	22
7	Diphenylamine (0.2 mol%)	EtOH	5	31
8	3DPAFIPN (0.3 mol%)	EtOH	5	97
9	—	EtOH	20	trace

^a^ Reaction conditions: several photocatalysts were combined with benzaldehyde (1.0 mmol), ethyl acetoacetate (1.0 mmol), and urea (1.5 mmol) at room temperature.

**TABLE 2:** The table for optimizing solvent and visible light in the synthesis of **4j** ^a^.

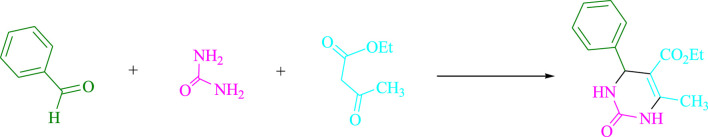				
Entry	Light source	Solvent (3 mL)	Time (min)	Isolated yields (%)
1	Blue light (5 W)	EtOAc	5	68
2	Blue light (5 W)	DMSO	25	29
3	Blue light (5 W)	toluene	25	26
4	Blue light (5 W)	H2O	6	71
5	Blue light (5 W)	EtOH	5	97
6	Blue light (5 W)	MeOH	8	59
7	Blue light (5 W)	—	8	46
8	Blue light (5 W)	CHCl3	30	17
9	Blue light (5 W)	CH3CN	5	75
10	Blue light (5 W)	THF	25	15
11	White light (5 W)	EtOH	5	82
12	Green light (5 W)	EtOH	5	90
13	Blue light (3 W)	EtOH	5	89
14	Blue light (7 W)	EtOH	5	97
15	—	EtOH	20	trace

^a^ Reaction conditions: a mixture of urea (1.5 mmol), benzaldehyde (1.0 mmol), and ethyl acetoacetate (1.0 mmol) was added to 0.2 mol% 3DPAFIPN.

**TABLE 3:** Using 3DPAFIPN, a halogenated dicyanobenzene-based photosensitizer, 3,4-dihydropyrimidin-2-(1*H*)-one/thione derivatives be produced.

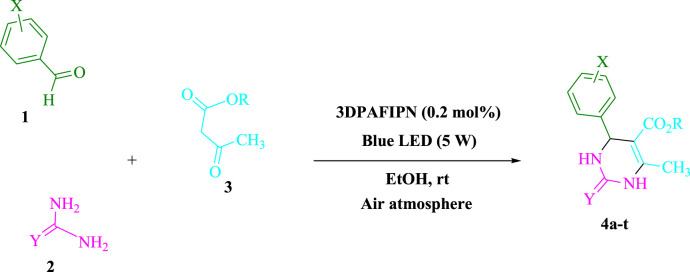			
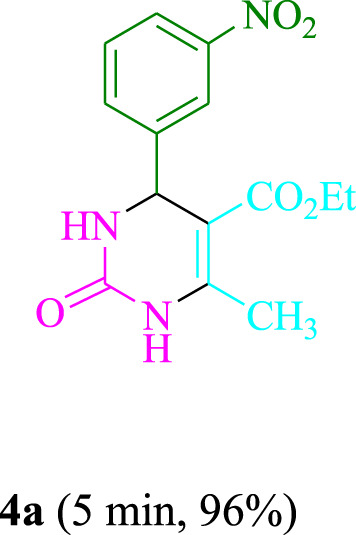	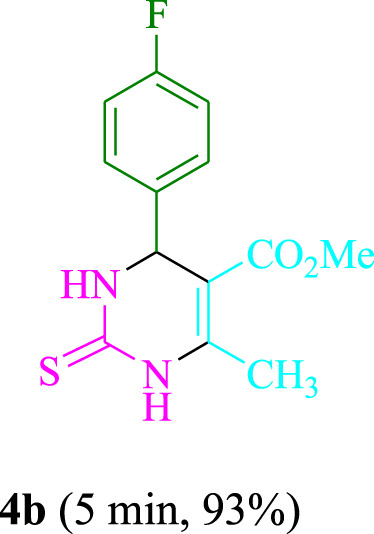	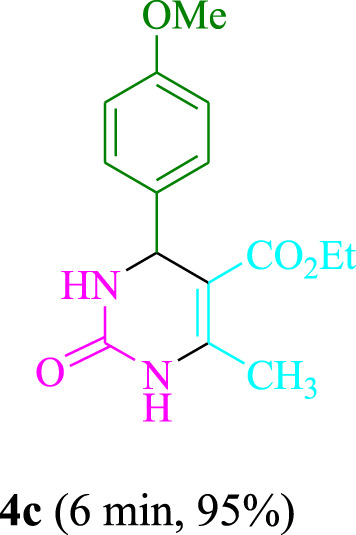	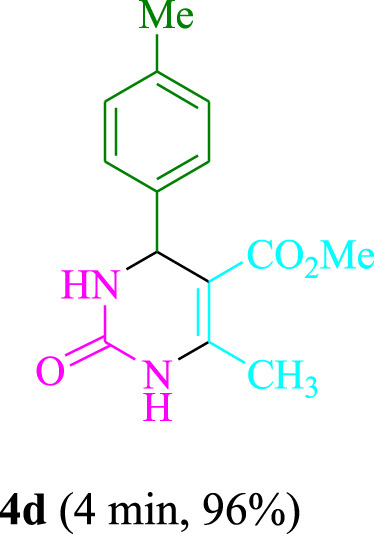
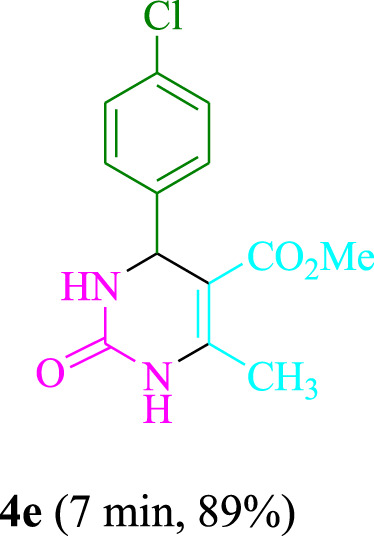	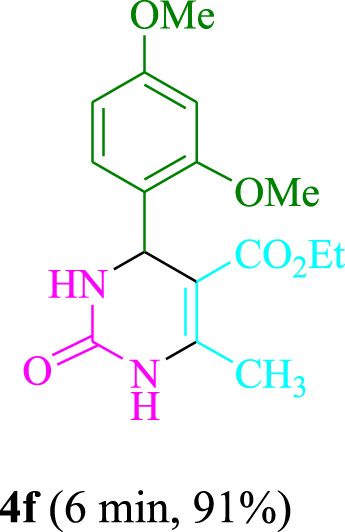	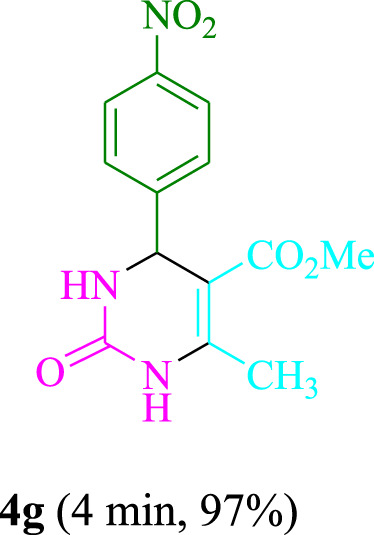	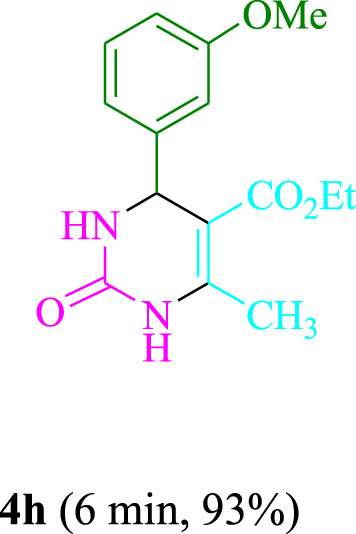
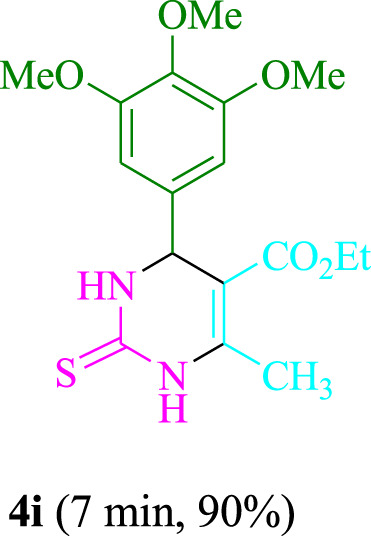	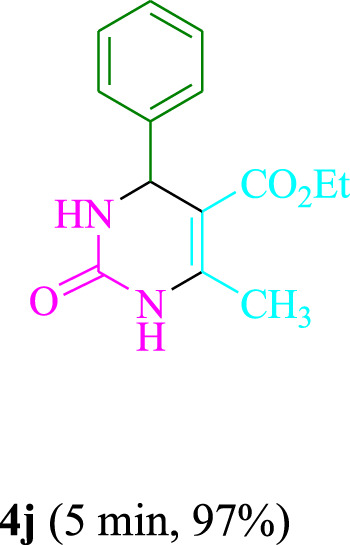	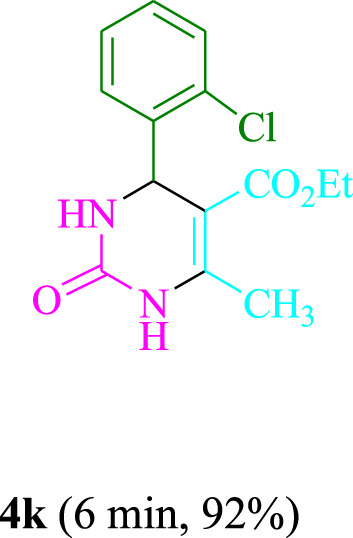	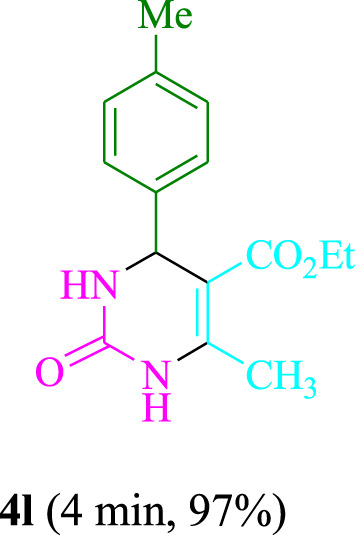
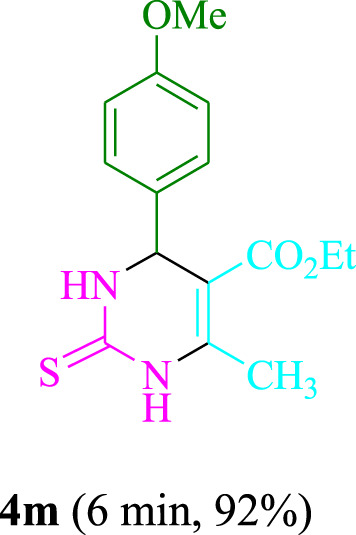	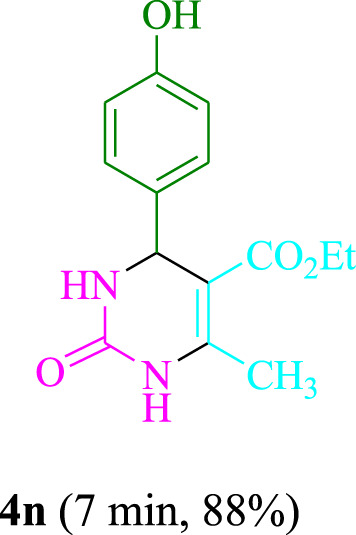	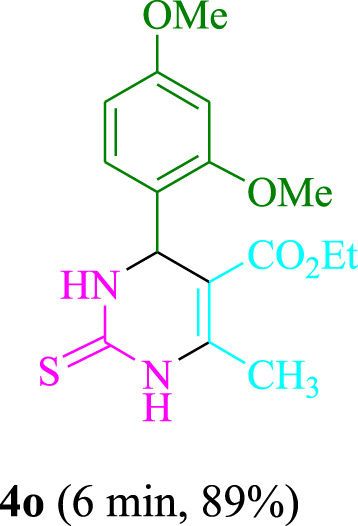	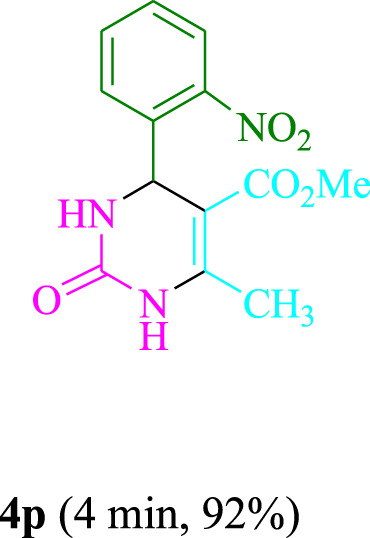
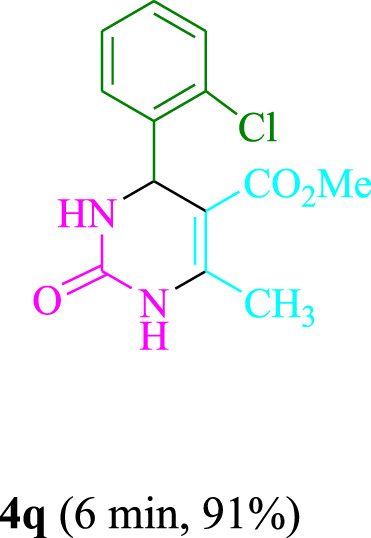	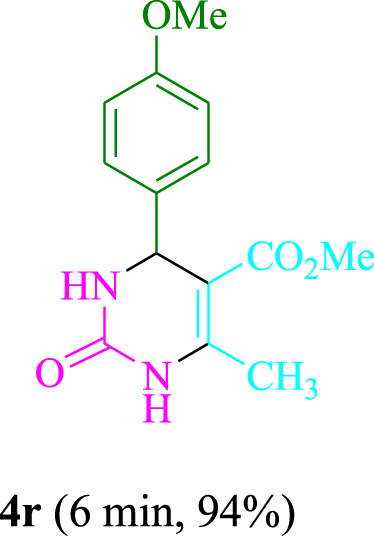	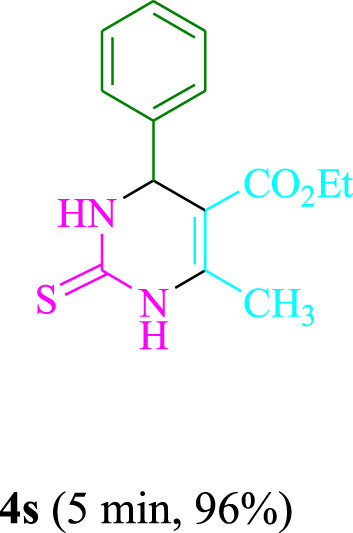	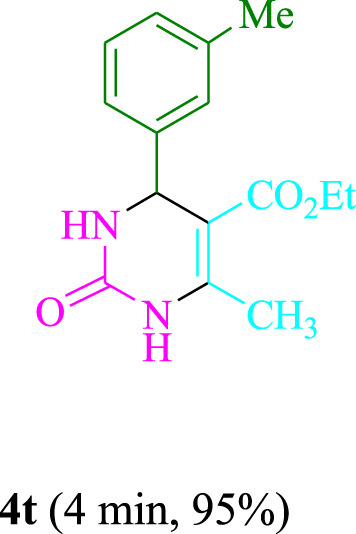

Table 4 presents the turnover number (TON) and turnover frequency (TOF) as objective value measures. Yield/Amount of Catalyst (mol) and Yield/Time/Amount of Catalyst (mol) are two distinct forms of yield that are commonly expressed as TON and TOF, respectively, in academic literature. The catalyst performance may be enhanced by higher turnover number (TON) and turnover frequency (TOF) values as they need less catalyst to provide the required yields. A TOF of 97 and a TON of 485 are considered high values for **4j**. In relation to **4s**, a TON of 480 is likewise regarded as high, although a TOF of 96 is deemed excessive. The aim of the inquiry was to reduce reaction times, increase production, and utilize the fewest amount of catalysts.

**TABLE 4:** The following calculated were used to determine the turnover frequency (TOF) and turnover number (TON).

Entry	Product	TON	TOF	Entry	Product	TON	TOF
1	4a	480	96	11	4k	460	76.6
2	4b	465	93	12	4l	485	121.2
3	4c	475	79.1	13	4m	460	76.6
4	4d	480	120	14	4n	440	62.8
5	4e	445	63.5	15	4o	445	74.1
6	4f	455	75.8	16	4p	460	115
7	4g	485	121.2	17	4q	455	75.8
8	4h	465	77.5	18	4r	470	78.3
9	4i	450	64.2	19	4s	480	96
10	4j	485	97	20	4t	475	118.7

### Gram-scale synthesis

The main emphasis of current research is whether the aforementioned chemicals can be produced on a gram-scale for use in pharmaceutical (R&D) procedures. In one experiment, 50 mmol of 4-methoxybenzaldehyde, 75 mmol of thiourea, and 50 mmol of ethyl acetoacetate were utilized. To retrieve the final product, a standard filtering process was applied after the 6-min reaction period. The ^1^HNMR spectroscopy results show that the chemical compound in question exhibits a high degree of spectroscopic purity.

### Control experiments

**SCHEME 2:**
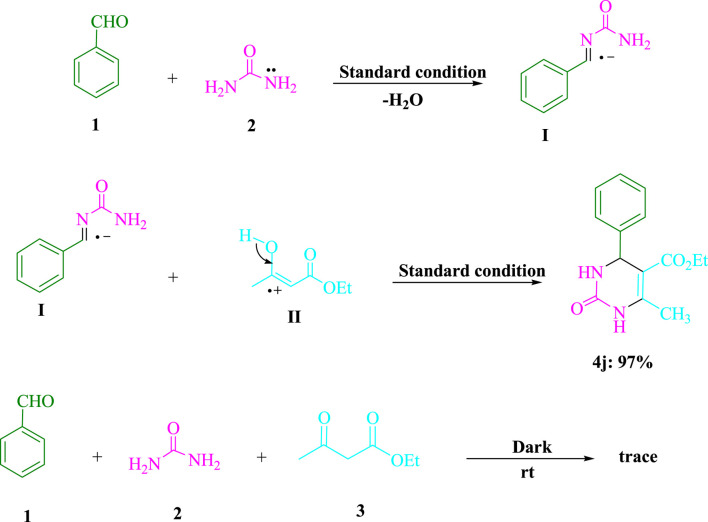
To comprehend the condensations of urea (**2**, 1.5 mmol), ethyl acetoacetate (**3**, 1.0 mmol), and benzaldehyde (**1**, 1.0 mmol), significant control tests are conducted.

**SCHEME 3:**
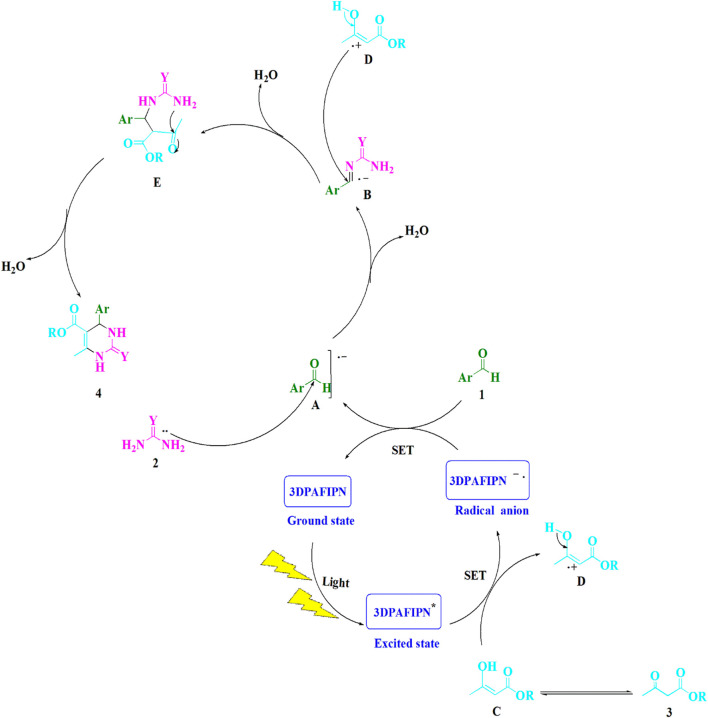
This is a detailed illustration of the synthetic procedure that yields 3,4-dihydropyrimidin-2-(1*H*)-one/thione derivatives.

Scheme 2 displays the outcomes of the control experiments used to elucidate the process utilizing the visible-light-induced. The synthesis of benzylideneurea (**I**) in the first stage and its condensation with ethyl acetoacetate (**3**) in the second are considered to be the two steps of the Biginelli reaction. Under standard circumstances (3DPAFIPN in EtOH under blue LED), benzaldehyde (**1**) and urea (**2**) were condensed by reducing H_2_O to produce benzylideneurea (**I**). As a consequence, in 97% of reactions between the iminium intermediate (**I**) and cation radical (**II**), under normal conditions, the expected product **4j** was generated. Even though the reaction was carried out in total darkness, there was still a trace of product **4j** created. The results of this experiment indicate that Scheme 3 presents a convincing and rational chemical process.

### The suggested mechanism

Scheme 3 provides a thorough description of the suggested methodology. Using single-electron transfer (SET) processes, the cyanoarene organic dye 3DPAFIPN has been utilized to develop photocatalytic reactions that use visible light energy as a sustainable resource. Utilizing visible light expedites the procedure. The ground-state 3DPAFIPN and the intermediate (**A**) regenerate as a result of the electron transfer (ET) activity between the arylaldehydes (**1**) and the 3DPAFIPN radical anion. A reactive iminium intermediate (**B**) is formed when this radical anion (**A**) is added nucleophilically to urea/thiourea (**2**). The single-electron transfer (SET) technique is utilized to enhance 3DPAFIPN^*^, which is produced by visible light, and produce the cation radical (**D**). The iminium intermediate (**B**) is attacked by the cation radical (**D**), leading to the formation of the cyclized dehydrated (**4**).

### Comparison of the catalytic activity of different catalysts with 3DPAFIPN

Table 5 compares how well various catalysts work to encourage the synthesis of 3,4-dihydropyrimidin-2-(1*H*)-one/thione derivatives. The process in question precipitates rapid chemical changes without generating any waste by using tiny quantities of photocatalyst. When there are quantifiable light wavelengths present, this approach can be used. At multigram scales, atom-economical processes are very efficient and have a big impact on the industrial domain.

**TABLE 5:** The results of assessing the various catalysts’ catalytic efficacy for the synthesis of **4j** ^a^.

Entry	Catalyst	Conditions	Time/Yield (%)	References
1	Bakers^,^ yeast	Room temperature	1,440 min/84	Kumar and Maurya (2007)
2	Hydrotalcite	Solvent-free, 80°C	35 min/84	Lal et al. (2012)
3	[Al(H_2_O)_6_](BF_4_)_3_	MeCN, Reflux	1,200 min/81	Litvic et al. (2010)
4	Cu(BF_4_)_2_.xH_2_O	Room temperature	30 min/90	Kamal et al. (2007)
5	[Btto][*p*-TSA]	Solvent-free, 90°C	30 min/96	Zhang et al. (2015)
6	triethylammonium acetate	Solvent-free, 70°C	45 min/90	Attri et al. (2017)
7	saccharin	Solvent-free, 80°C	15 min/88	Mohamadpour et al. (2016)
8	caffeine	Solvent-free, 80°C	25 min/91	Mohamadpour and Lashkari (2018)
9	3DPAFIPN	Blue LED, EtOH, rt	5 min/97	This work

^a^ The synthesis requires three ingredients: urea, ethyl acetoacetate, and benzaldehyde.

## Conclusion

We have green photosynthesized 3,4-dihydropyrimidin-2-(1*H*)-one/thione derivatives from arylaldehydes, *β*-ketoesters, and urea/thiourea by means of the radical-induced Biginelli reaction. In the current work, a new halogenated dicyanobenzene-based photosensitizer; 3DPAFIPN was employed as a donor-acceptor (D-A) photocatalyst. It works by causing a sequence of electron transfers that are triggered by visible light. Blue light-emitting diode (LED) technology has been demonstrated to generate a sustained energy-generating mechanism at room temperature and in an air environment when used in an ethanol medium. The suggested method has significant advantages for the field of chemical synthesis. Fast reaction times, the removal of hazardous solvents, higher product yields, streamlined reaction mechanisms, and the utilization of a sustainable energy source are some of these benefits. The separation method does not need chromatography. By preserving the result, it is possible to accelerate a multigram-scale reaction of model substrates. As a result, the method may be used in an environment that promotes long-term ecological and financial sustainability.

## Data Availability

The original contributions presented in the study are included in the article/Supplementary Material, further inquiries can be directed to the corresponding authors.
