# Supplementary material for: Halogenated dicyanobenzene-based photosensitizer (3DPAFIPN) as a thermally activated delayed fluorescence (TADF) used in gram-scale photosynthesis 3,4-dihydropyrimidin-2-(1H)-one/thione derivatives via a consecutive visible-light-induced electron-transfer pathway
Source: Front Chem. 2024 Mar 1;12:1361266. doi: 10.3389/fchem.2024.1361266 (PMC10943697; doi:10.3389/fchem.2024.1361266)
Supplement: Supplementary file 1 [file DataSheet1.pdf]

Supporting Information

Halogenated dicyanobenzene-based photosensitizer (3DPAFIPN) as a thermally activated delayed fluorescence (TADF) used in gram-scale photosynthesis 3,4-dihydropyrimidin-2-(1*H*)-one/thione derivatives via a consecutive visible-light-induced electron-transfer pathway

Farzaneh Mohamadpour[a,*], Ali Mohammad Amani[a,*]

[a]Department of Medical Nanotechnology, School of Advanced Medical Sciences and Technologies, Shiraz University of Medical Sciences, Shiraz, Iran

Corresponding authors email: f_mohamadpour@sums.ac.ir; mohamadpour.f.7@gmail.com; amani_a@sums.ac.ir, aliamani@sums.ac.ir

Table S1. 3DPAFIPN-catalyzed synthesis of 3,4-dihydropyrimidin-2-(1*H*)-one/thione derivatives[a]

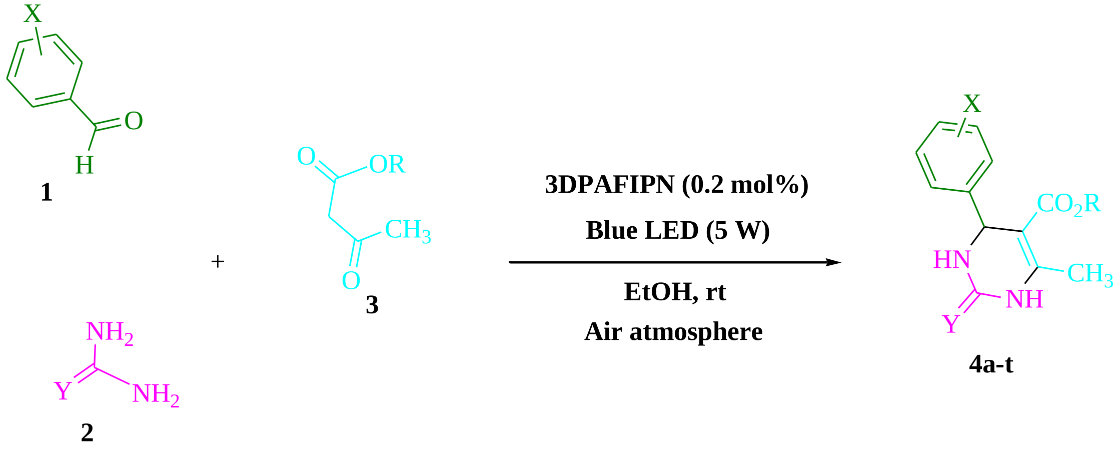

Entry	Product	M.p. (°C)		Entry	Product	M.p. (°C)	
		Found	Reported			Found	Reported
1	**4a**	224-226	225-229 [37]	11	**4k**	222-224	220-223 [26]
2	**4b**	212-214	210-212 [35]	12	**4l**	213-215	216-217 [41]
3	**4c**	200-202	202-203 [28]	13	**4m**	148-150	150-152 [29]
4	**4d**	220-222	220-222 [42]	14	**4n**	232-235	230-232 [30]
5	**4e**	207-209	205-206 [33]	15	**4o**	161-163	163-164 [41]
6	**4f**	207-209	210-211 [41]	16	**4p**	276-278	274-277 [31]
7	**4g**	213-215	214-216 [29]	17	**4q**	251-253	248-252 [26]

8	**4h**	202-204	205-206 [27]	18	**4r**	185-187	186-189 [31]
9	**4i**	197-199	195-197 [35]	19	**4s**	209-212	208-210 [29]
10	**4j**	201-202	200-202 [29]	20	**4t**	204-206	203-206 [37]

[a]Reaction conditions: at room temperature in EtOH (3 mL), urea/thiourea (**2**, 1.5 mmol), ethyl/methyl acetoacetate (**3**, 1.0 mmol), and arylaldehyde derivatives (**1**, 1.0 mmol) were stirred with 3DPAFIPN (0.2 mol%) and blue light (5 W).

More Discussion

According to the current investigation's results, 3DPAFIPN stands out from other catalysts due to its high yield, quick reaction time, and superior TON and TOF levels. Moreover, a critical component of 3DPAFIPN's use in industrial applications is the minimum amount of catalyst required in the reaction (0.2 mol%). This feature makes it an economical yet environmentally friendly option for industrial applications. In addition, higher yields are associated with higher levels of catalytic efficiency and higher Turnover Number (TON) and Turnover Frequency (TOF) quantitative representations. The higher these values are, the more effective the catalytic activity. As evidenced by its remarkable TON (turnover number) of 485 and TOF (turnover frequency) of 97, 3DPAFIPN exhibits a remarkably high degree of catalytic activity for the synthesis of **4j**, according to the study.

The results of the experiment show that 3DPAFIPN uses the visible light to quickly move from the ground state to the excited state. The faster chemical reaction rate could be caused by the combined effects of being exposed to visible light and 3DPAFIPN. Because of their advantageous

economic and environmental qualities, organic photocatalysts like 3DPAFIPN have been demonstrated to be a viable substitute for other redox photocatalysts. A comprehensive analysis was conducted on the photochemical behavior of 3DPAFIPN upon stimulation by visible light. These studies demonstrated that 3DPAFIPN has a rapid inter-system transition to the lowest energy triplet state. The remarkable light-absorbing properties of 3DPAFIPN, along with its inherent synthesis efficiency and potential, make it a promising compound for numerous visible light-catalyzed chemical reactions.

Using blue light (5 W) as a renewable energy source at room temperature, 3 mL EtOH as green solvent, 3DPAFIPN (0.2 mol%) as a halogenated dicyanobenzene-based photosensitizer, 3,4-dihydropyrimidin-2-(1*H*)-one/thione derivatives synthesis have all been studied in the current work. With an average of 93.15%, the yields range from 88 to 97%. Reaction time also ranges from 4 to 7 minutes, with an average of 5.45 minutes.

This study is a suitable and effective option for industrial purposes due to the following advantages:

1) The present work uses a novel halogenated dicyanobenzene-based photosensitizer in conjunction with blue light to facilitate the synthesis of 3,4-dihydropyrimidin-2-(1*H*)-one/thione derivatives in an environmentally sustainable manner through the well-known Biginelli reaction. Based on the results, the method shows promise as a cohesive strategy when the response conditions are straightforward and effective. Advocates of sustainable chemistry find great appeal in the combination of renewable energy sources and halogenated cyanoarene-based donor-acceptor (D-A) photocatalyst in an ethanol solution because of its remarkable speed and yield.

2) There is evidence from this study to bolster the claim that there is a notable level of uniformity in the distribution of crop yields, with an average of 93.15% and a range of 88-97%. With a time frame of 4-7 min and an average of 5.45 min, the chronological parameters associated with the process, in contrast, exhibit signs of rapid kinetics. One key finding relevant to the discussion at

hand is that this approach keeps a wide variety of donating and withdrawing functional groups, which in turn encourages the regular attainment of high yields. No matter what the substituent moieties are made of, the reaction will still be susceptible to rationalization.

3) The compound's pharmaceutical significance is underscored by its production scale, which can reach up to multigram scales. Moreover, noteworthy attributes comprise an optimized experimental setup, ability to react with a wide range of substrates, economy, simple work-up methods that eliminate labor-intensive separation processes, and low production of organic waste per conversion unit.

Spectroscopic data

5-Methoxycarbonyl-6-methyl-4-(4-nitrophenyl)-3,4-dihydropyrimidin-2(1H)-one (4g)

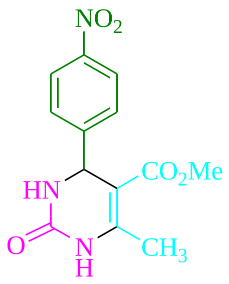

Yield: 97%; M.p. 213-215 °C; ^{1}HNMR (300 MHz, DMSO-d$_6$): 2.32 (3H, s, CH$_3$), 3.47 (3H, s, OCH$_3$), 5.24 (1H, s, H$_{benzylic}$), 7.23 (2H, d, *J*= 11.2 Hz, H$_{Ar}$), 7.46 (2H, d, *J*= 11.2 Hz, H$_{Ar}$), 7.81 and 9.27 (2H, 2s, 2NH).

5-Ethoxycarbonyl-6-methyl-4-(4–methoxyphenyl)-3,4-dihydropyrimidin-2(1H)-thione (4m)

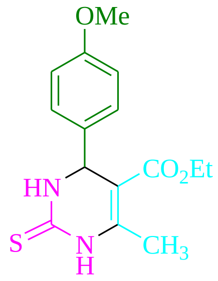

Yield: 92%; M.p. 148-150 °C; ^{1}HNMR (300 MHz, DMSO-d$_6$): 1.13 (3H , t, *J*= 9.6 Hz, $\underline{CH_3}CH_2$), 2.29 (3H, s, CH_3), 3.74 (3H, s, OCH_3), 4.03 (2H, q, *J*=9.6 Hz, CH_2O), 5.15 (1H, s, $H_{benzylic}$), 6.77 (2H, m, H_{Ar}), 6.87 (1H, m, H_{Ar}), 7.28 (1H, t, *J*=9.6 Hz, H_{Ar}), 9.66 and 10.37 (2H, 2s, 2NH).

5-Methoxycarbonyl-6-methyl-4-(2-chlorophenyl)-3,4-dihydropyrimidin-2(1H)-one (4q)

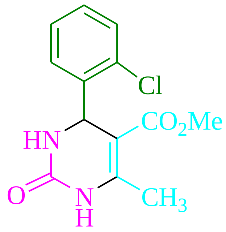

Yield: 91%; M.p. 251-253 °C; ^{1}HNMR (300 MHz, DMSO-d$_6$): 2.24 (3H, s, CH_3), 3.52 (3H, s, OCH_3), 5.28 (1H, s, $H_{benzylic}$), 7.15-7.22 (2H, m, H_{Ar}), 7.31-7.36 (2H, m, H_{Ar}), 7.76 and 9.17 (2H, 2s, 2NH).

5-Methoxycarbonyl-6-methyl-4-(4-methoxyphenyl)-3,4-dihydropyrimidin-2(1H)–one (4r)

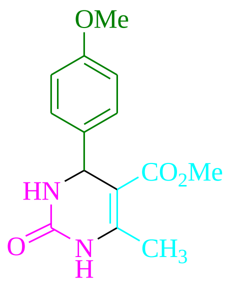

Yield: 94%; M.p. 185-187 °C; ^{1}HNMR (300 MHz, DMSO-d$_6$): 2.25 (3H, s, CH_3), 3.53 (3H, s, OCH_3), 3.73 (3H, s, OCH_3), 5.09 (1H, s, $H_{benzylic}$), 6.88 (2H, d, *J*= 8.8 Hz, H_{Ar}), 7.14 (2H, d, *J*= 8.8 Hz, H_{Ar}), 7.71 and 9.20 (2H, 2s, 2NH).
